# Patient Perspectives on the Participation of Neurosurgery Resident Physicians in Their Care

**DOI:** 10.7759/cureus.6880

**Published:** 2020-02-04

**Authors:** Brian Fiani, Alessandra Cathel, Mohammad Arshad, Hamid Hadi, Yasir R Khan, Syed A Quadri, Anthony Alastra, Javed Siddiqi

**Affiliations:** 1 Neurosurgery, Desert Regional Medical Center, Palm Springs, USA; 2 Neurosurgery, Massachusetts General Hospital, Harvard Medical School, Boston, USA

**Keywords:** patient, patient's perspective, neurosurgery, resident, feedback, survey

## Abstract

Introduction

Surgical residents play vital roles in day-to-day patient care as well as in the operating room. However, there is a paucity of literature regarding patients’ perspectives on neurosurgical residents and their participation in their care. This current study investigates the knowledge, attitudes, and beliefs of patients regarding neurosurgical residents and their involvement in their healthcare process.

Methods

Patients older than 18 years who had undergone brain or spine surgery were requested to complete a survey questionnaire. The 7-point Likert scale response ranging from “strongly agree”, “agree”, “more or less agree”, “undecided”, more or less disagree”, “disagree” to “strongly disagree” was used. The primary objective was to assess patient understanding and attitudes towards resident participation in surgical and medical care within the specialty of neurosurgery. The duration of the study was eight months. Patients having prior exposure to an informed-consent procedure by a neurosurgery team within a year prior to this study were excluded. Data were analyzed using Student’s t-test, one-way analysis of variance (ANOVA), and standard averaging of responses.

Results

Fifty-one patients who had undergone elective surgery participated in the study survey. The majority of these respondents were between the ages of 46 and 60 years. Most of the responses were similar across gender and different age groups for most of the questions on the Likert scale questionnaire. Overall, when asked to assess their comfort level in medical and surgical care participation by residents, patients responded positively (strongly agree: 80.4%; agree: 92.2%). Patients also either disagreed or strongly disagreed (76%) about residents lacking medical knowledge. Patients overwhelmingly disagreed (91.5%) when asked if residents were unprofessional. In addition, 72.5% of the patients were able to accurately define a resident’s role.

Conclusion

Well-formatted surveys can offer a convenient route for patients to provide objective as well as subjective feedback. The results indicate that patients had an overall positive attitude toward having residents involved in their care. These trends also indicate that patients knew the role that residents played in their healthcare process and they were comfortable with them doing so. Further studies may expand the trial to include a larger number of patients, as well as other specialties, to expand the scope of the study. Patient survey questionnaires could be thought of as a useful tool by the Accreditation Council for Graduate Medical Education (ACGME) to incorporate as part of the evaluation process of resident physicians.

## Introduction

The Accreditation Council of Graduate Medical Education (ACGME) has developed accreditation guidelines for residency programs to follow so that there is both an intra- and inter-specialty standard of training throughout the United States [1]. The ACGME defines a residency program as a structured educational activity comprising a series of clinical or other learning experiences in graduate medical education, which is designed to prepare physicians to enter the unsupervised practice of medicine in a primary specialty [2]. In order to achieve standardization of training across all specialties, the ACGME has incorporated six core competencies: clinical knowledge, patient care, systems-based practice, practice-based learning, professionalism, and interpersonal communication [3].

Emphasis on patient safety and medical error reduction has characterized the landscape of healthcare in the United States. Patients often desire the most experienced physician, especially in the intra-operative setting where iatrogenic complications are more likely to lead to morbidity or mortality [4]. Although it has been demonstrated that resident participation in surgical cases, particularly neurosurgical cases, does not increase morbidity or mortality, many patients continue to be apprehensive about having residents involved in their surgeries [5,6]. Patients undergoing surgery are frequently under significant stress. The patients’ trust of their surgeon is the cornerstone of reassurance during preoperative anxiety [7,8]. Many patients want to be informed preoperatively as to who the residents are and what their roles will be during their surgery. Learning about this information postoperatively may harm the patient-physician relationship [8,9]. Lack of appropriate understanding of resident training process by patients may create misconceptions about resident physicians and, ultimately, decrease trust in the healthcare system [8,10]. Preoperative psychological studies have shown that patients who have a stronger sense of control in regards to their surgical care also experience accelerated recovery [8]. A patient’s understanding of their physician’s level of education and role in their healthcare has been found to be important to the majority of patients. At the same time, there are reports suggesting that only 65% of surgical patients are aware if their surgeon is a trainee or not [10,11]. There are other reports that inform that the majority of patients believed residents to be medical students [11].

There is a paucity of literature regarding patients’ perspectives on neurosurgical residents and their participation in their care [8]. The current study investigates the knowledge, attitudes, and beliefs of patients regarding neurosurgical residents and their involvement in the healthcare process.

## Materials and methods

Patients undergoing elective brain or spine surgery were requested to complete an anonymous survey questionnaire to elicit the level of patient knowledge and understanding of the role of a neurosurgery resident in their care. The patient perspective analyzed involved their level of comfort in a teaching-hospital environment. The process for the patients’ inclusion in this study went as follows: initial presentation to the neurosurgery outpatient clinic office by scheduled appointment, surgical scheduling, and subsequent surgical intervention. The questionnaires were distributed by the residents to the patients upon discharge from the hospital. The participants were asked to define terms such as “resident” and “assistance in surgery” as well as to mention the level/position of the medical practitioner from whom they received the majority of the details of their surgical plan. The options provided in the 7-point Likert scale response ranged from “strongly agree”, “agree”, “more or less agree”, “undecided”, more or less disagree”, “disagree” to “strongly disagree” (see Appendices). Our primary objective was to assess the patients’ understanding and attitudes.

The duration of the study was eight months, and a total of 51 surveys were collected in this cross-sectional, anonymous survey study. The inclusion criteria for this study were as follows: (i) patients who were willing to participate and (ii) patients older than 18 years who had documented neurosurgical diagnoses and had undergone a neurosurgical procedure for their brain or spine. The exclusion criteria included patients who had prior exposure to an informed-consent procedure by a neurosurgery team in the last one year, so as to avoid bias based on another event. Descriptive statistics of the responses were calculated. Student’s T-test and one-way analysis of variance (ANOVA) were employed to assess the associations. A p-value of 0.05 was considered statistically significant for all the tests. All the analyses were performed using Microsoft Excel (Microsoft Corporation, Redmond, WA).

## Results

**Figure 1:**
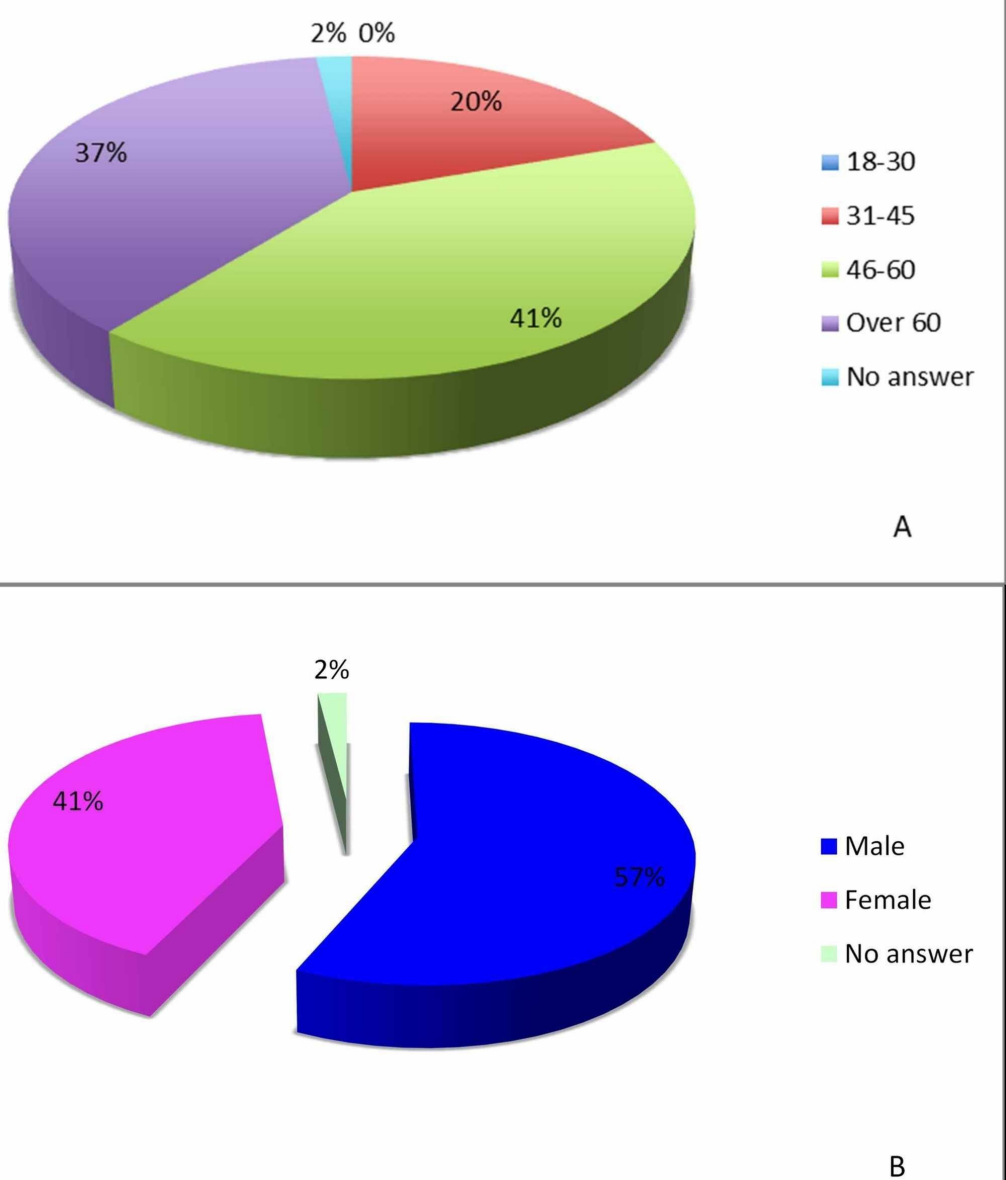
Characteristics of the survey participants A: characteristics pertaining to age; B: characteristics pertaining to gender

A total of 56 patients participated in the study survey. All of them had undergone elective surgeries at Desert Regional Medical Center, Palm Springs, CA. Five patients had had previous surgeries and were excluded. A total of 51 patients [29 males (57%), 21 females (42%), and one who preferred not to reveal gender (1%)] were included in the final analyses (Figure 1).

**Figure 2:**
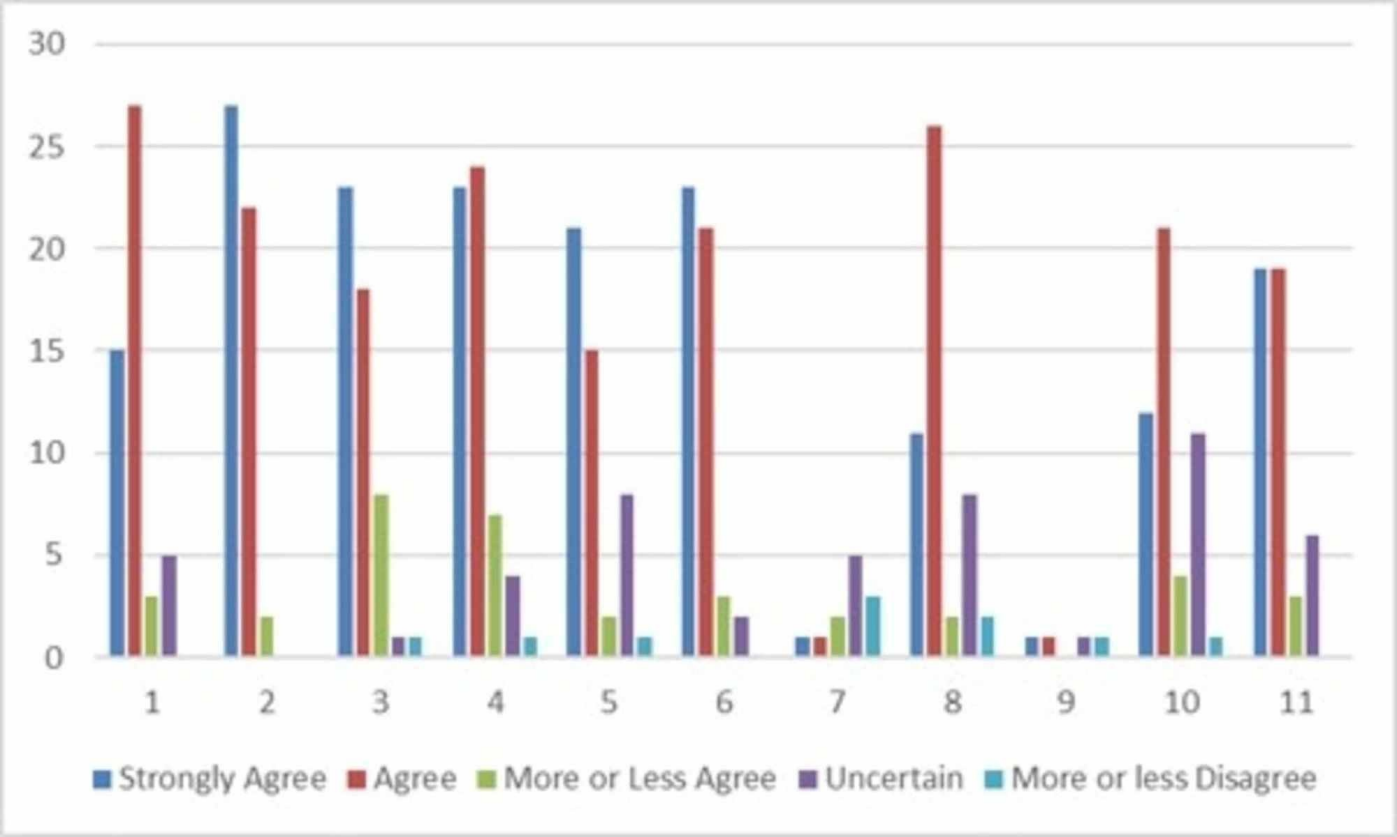
Responses to the survey The horizontal axis corresponds to question numbers provided in the survey

When tabulating the responses to the 7-point Likert scale, the majority of responses were similar across the age groups and gender for all questions (Figure 2).

**Figure 3:**
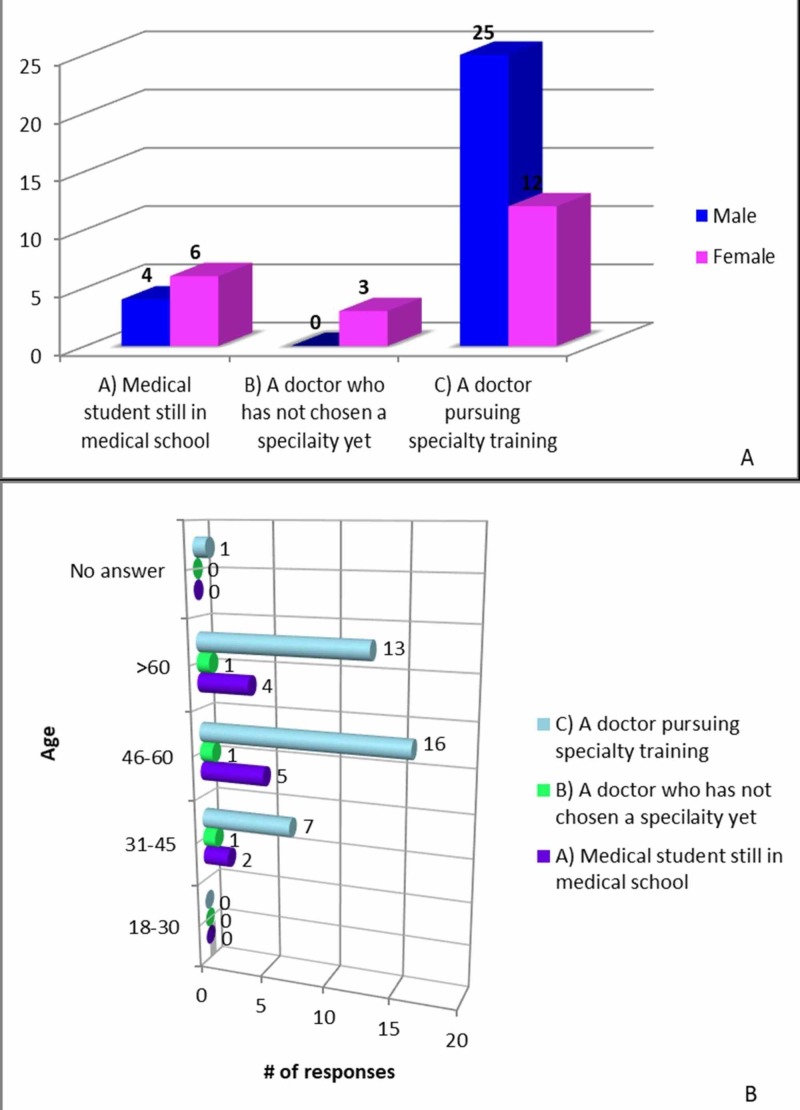
Patient responses to “definition of resident” A: responses by gender; B: responses by age group

With regard to patients’ perspective on residents lacking medical knowledge, 41% “strongly disagreed” and 35% “disagreed”. Also, the majority of the patients either “strongly disagreed” or “disagreed” when questioned if residents were unprofessional (70 and 21.5%, respectively). The patients’ comfort level was also assessed. Overall, patients were comfortable helping residents become better surgeons by allowing their participation in all aspects of their care (45.1% “strongly agreed” and 35.3% “agreed”). A similar trend was seen with respect to comfort level regarding residents’ surgical participation (45.1% “strongly agreed” and 47.1% “agreed”). When asked to give the most accurate definition of a resident physician, most patients selected the response “a doctor pursuing specialty training” (72.5%) (Figure 3).

**Figure 4:**
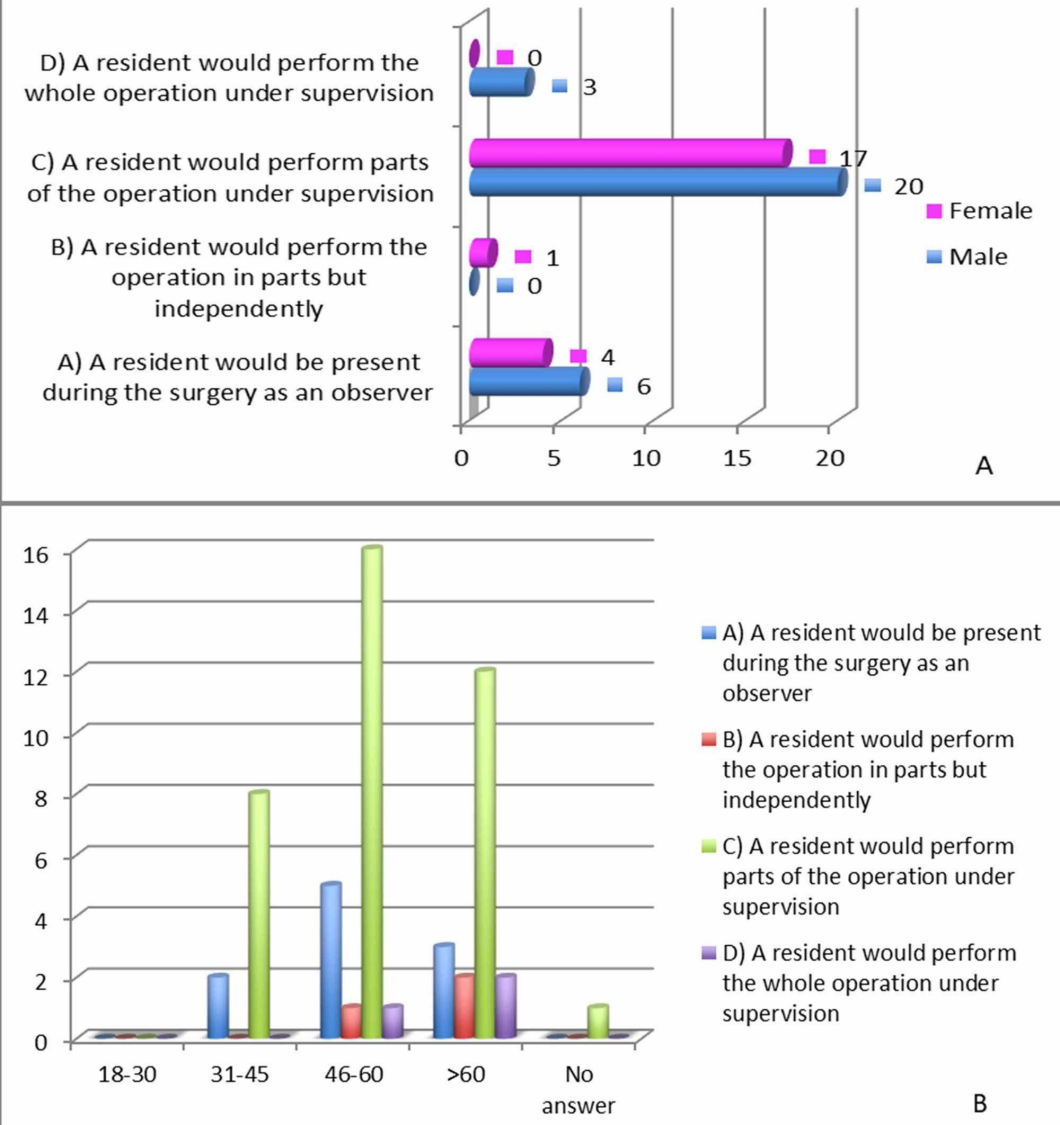
Patient responses to “what does assisting in surgery mean” and “the role of the resident during surgery” A: responses by gender; B: responses by age group

The responses to this question were similar among all genders and across the age groups (p-value: 0.66 and 0.21, respectively). A similar trend was seen in response to the question “what does assisting in surgery mean” (p: 0.16 and 0.25, respectively), where the majority (72.5%) of the patients responded that “a resident would perform parts of the operation under supervision” (Figure 4).

**Figure 5:**
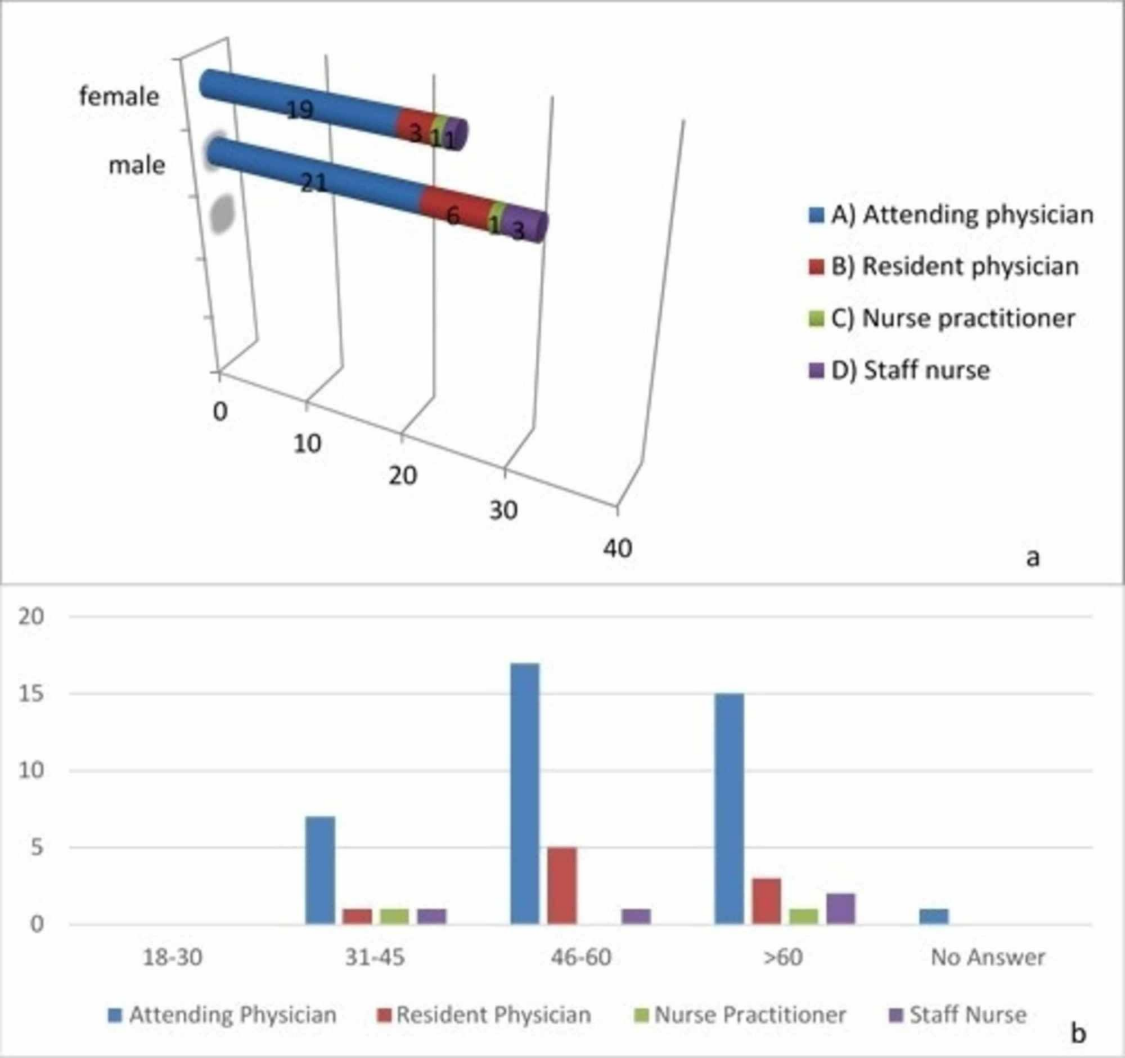
Patient responses to “who provided the most information regarding surgery” A: responses by gender; B: responses by age group

However, for the question “who provided the most information regarding surgery”, the majority of the patients (78.4%) chose “the attending physician” as their answer (p: 0.07). However, the difference in response by age groups for this question was not significant (p; 0.32) (Figure 5).

## Discussion

The primary objective of our study was to assess the patient understanding and attitudes towards resident physicians’ participation in surgical and medical care within the neurosurgical specialty. Other studies have been performed over the years to learn what patients know and how they feel about resident engagement in their healthcare in general. However, to the best of our knowledge, no studies have investigated the patient perspective on this matter pertaining specifically to neurosurgery resident physicians.

The distribution of paper copies of the survey questionnaire to the participants turned out to be a successful tool to measure patient feedback. This method enabled us to directly gain insight from patients by physically handing them the questionnaire. The survey questions were easy to read and provided direct and quantifiable responses for analysis. Although the online survey method was a viable alternative, we hypothesized that it would elicit a worse response rate compared to the physical method due to patients having to voluntarily complete the survey and the extra effort required by them to access it online and complete it.

The method of using a survey technique also turned out to be a point of strength because the patients appreciated the opportunity to provide feedback. The survey also provided the opportunity of gaining insight into improving resident-patient interaction. Patients are seen during their healthcare process by multiple providers from the healthcare team, including nurses, nurse practitioners, resident physicians, and attending physicians. In this diverse setting, patients have the opportunity to develop a clearer understanding of the neurosurgical resident’s role in their care.

A Likert scale was used to easily calculate mean responses. Other statistical tests were employed but did not provide any results of statistical significance. These findings were expected since the gender and age characteristics were not anticipated to impact the responses. There were no statistical differences pertaining to responses for the most part among genders and age groups. However, there were trends and mean/average answers that were consistent across all groups. The majority of the patients in all groups chose “attending neurosurgeon” as the answer (male: 21, female: 19; 78.4%) when asked about the major source of information regarding their care and surgery. This result was unexpected given that the resident typically spends more time with the patient, including on reviewing surgical risks, benefits, and alternatives when consulting with the patients. However, this response could be explained by the fact that the attending neurosurgeon is the most experienced and authoritative figure of a healthcare team, and, therefore, it is likely that they were able to communicate with patients more effectively and educate and address their concerns in less time than the resident physicians. To keep the responses anonymous, the residents were not allowed to ask patients about their answers while the questionnaires were being distributed.

Similar trends were noted for questions relating to both resident definition and assistance in surgery. The bulk of the patients responded with the same answer choice: a “doctor pursuing specialty training” and “a resident would perform parts of the operation under supervision,” respectively (72.5% and 72.5%, respectively). From this data, one can infer that the responsibilities and duties of a resident were clearly described to the patients.

Clear trends were noted when evaluating the responses from the Likert scale questions as well (Figure 4). For this study, the most important of those pertained to patients’ comfort level with resident involvement in their care, actual involvement a resident plays in their care, whether the resident involvement resulted in better care, and the patients’ perceptions regarding residents’ professionalism and medical knowledge. The majority of the patients (23 of 51) “strongly agreed” that they were comfortably in residents’ participation in their care. When this question was tailored specifically to surgical involvement, 24 of 51 patients “agreed” and 23 of 51 “strongly agreed” that they were comfortable with resident participation in their surgery. Thus, 47 of the 51 patients’ responded positively to resident involvement, relating to both care in general and specifically to surgical care. When asked if residents lacked medical knowledge or were unprofessional, the majority of the patients chose “strongly disagree” (21 and 36 patients, respectively). Eighteen patients “disagreed” that the residents lacked medical knowledge, and 11 patients “disagreed” that residents behaved unprofessionally. This further demonstrates that the overall feeling towards residents was positive in nature.

As highlighted, all survey answers demonstrated a clear trend, though no question resulted in statistical significance. This is likely due to the low power of the study (5%), as opposed to the typical goal of 80%. One possible reason for this could be the small sample size. Some surveys had to be discarded as they were not filled out completely or due to patients having a neurosurgery encounter within one year. Many elective cranial cases were missed opportunities, as they were not always offered participation. Additionally, some patients did not elect to participate in the study.

We were pleased to see that the overall patient perception toward resident participation was positive. The major strengths of the study were the formulation of the patient survey, the design of the survey, and the method adopted for gathering data. Much time was dedicated to framing questions in such a manner that biased responses would not creep in. This was accomplished by including both positively and negatively angled questions. The survey questions were deemed user-friendly and easy to complete (with a multiple-choice format), which was a goal of the investigators from the inception of the project itself.

A major limitation of this study centered on the patient population. A hospital’s patient population is largely determined by the location of a hospital, the community which it serves, and its specific designations for specialty care. The hospital of this study is designated as a level-2 trauma center. As a trauma center, the neurosurgery service sees more trauma patients than elective ones. The survey was specifically designated for elective patients, while trauma patients were excluded. The reason for this exclusion was that trauma patients did not voluntarily choose to seek out medical attention from our institution. Additionally, it was assumed that trauma patients would not be in a position to complete a survey due to the severity of their condition. The low volume of elective patients meant that we had to prolong the study period as it took a considerably longer time to reach the goal of 50 completed surveys. The power of this study was calculated to be only 5%. Hence, for a more vigorous and result-oriented statistical analysis, the study population should be increased in similar studies in the future.

Improvement regarding future studies on this subject should focus predominantly on the survey distribution. To enhance the volume of surveys distributed and collected, there could be a single designated survey distributor. Alternatively, elective patients can be given peri-operative folders regarding their upcoming surgical procedure and could have the survey included in that folder for them to complete and bring to their follow-up appointments. A learning point from the study was the importance of patient introductions, which could also be a study improvement. The way in which a healthcare team member introduces himself or herself to a patient directly defines their role in the care of that patient from the patient perspective, thereby creating a better understanding of the role for the patient.

When comparing this study with others referenced, the overall trend of positivity was reinforced. In Petravick et al. (2007), the survey was conducted electronically, involving 200 patients and family members, and the residents were evaluated by their training experience. They found that comfort level increased as the residence experience increased with each post-graduate year [4]. This could be one area that future investigations could consider to include. In Cowles et al. (2001), which surveyed 200 general surgery patients, the majority felt comfortable with resident involvement and felt it was necessary to enhance their education and training [7]. Our patient population’s response was found to be similar to this prior study, with a similar desire for resident inclusion in their healthcare process and an appreciation for their involvement.

On the other hand, Knifed et al (2008) found that patients pooled from a group primarily undergoing craniotomies did not understand the role of a resident, but were nonetheless comfortable including them in their care [8]. In contrast, our study found that the role of the resident was well understood by the majority of the patients. From this finding, one may reasonably extrapolate that, over the past decade, patients have asked more questions, investigated on their own, or just overall become more knowledgeable with regard to the medical profession and the training process. With the advent of social media and the popularity of certain medical dramas, it could be hypothesized that pop culture has played a more prominent role than scholarly works in the improved understanding among patients that this study illustrated. This could be another avenue to be explored in future studies.

Our study demonstrates that, overall, the patients understood the resident role and deemed it a positive interaction. Expanding upon this study, one could investigate if there is a difference in perspective between patients undergoing elective surgery and those in the acute trauma setting. Including trauma patients into the study would also possibly change the overall age demographic to bi-modal distribution. Also, making the goals of the study and inclusion criteria clear would reduce the provider error. The scope of future studies could be further expanded by including more cranial cases. This would also provide an opportunity to evaluate whether patients undergoing cranial surgery perceive the resident involvement any differently compared to those undergoing spine surgery. Additionally, including more demographic information such as race and education level and adding more gender responses would broaden and enhance future studies.

## Conclusions

Our findings indicate that patients had an overall positive attitude toward having residents involved in their care. We can also infer that patients were well-informed about the role that residents played in their healthcare process and that they were generally comfortable with resident participation. Furthermore, they felt that residents had adequate knowledge and were professional in their conduct. We can conclude that the residency training process is beneficial for both patients and resident physicians. Further studies could expand the scope of the investigation by including trauma patients and increasing the survey population in terms of age groups. Honest patient feedback is an important tool to gain insight into various aspects of the healthcare process, and conducting well-formed surveys offers patients a convenient route to provide such feedback.

## Figures and Tables

**Figure 6:**
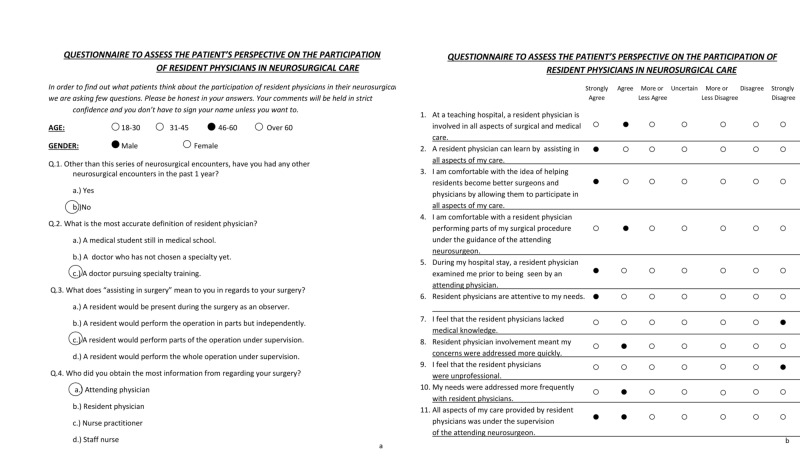
Questionnaire with most common answers highlighted

## Appendices

The survey used for the study
